# Peptide Signaling Pathways Regulate Plant Vascular Development

**DOI:** 10.3389/fpls.2021.719606

**Published:** 2021-09-03

**Authors:** Bingjian Yuan, Huanzhong Wang

**Affiliations:** ^1^Department of Plant Science and Landscape Architecture, University of Connecticut, Storrs, CT, United States; ^2^Institute for System Genomics, University of Connecticut, Storrs, CT, United States

**Keywords:** vascular development, peptide, cambium, signaling, Arabidopsis

## Abstract

Plant small peptides, including CLAVATA3/EMBRYO SURROUNDING REGION-RELATED (CLE) and Epidermal Patterning Factor-Like (EPFL) peptides, play pivotal roles in coordinating developmental processes through cell-cell communication. Recent studies have revealed that the phloem-derived CLE peptides, CLE41/44 and CLE42, promote (pro-)cambial cell proliferation and inhibit xylem cell differentiation. The endodermis-derived EPFL peptides, EPFL4 and EPFL6, modulate vascular development in the stem. Further, several other peptide ligands CLE9, CLE10, and CLE45 play crucial roles in regulating vascular development in the root. The peptide signaling pathways interact with each other and crosstalk with plant hormone signals. In this mini-review, we summtarize the recent advances on peptides function in vascular development and discuss future perspectives for the research of the CLE and EPFL peptides.

## Introduction

Intercellular communication and interaction are fundamentally important for plants to fine-tune growth and development in response to ever-changing environmental conditions (Kucukoglu and Nilsson, 2015). Secreted signaling peptides play crucial roles in regulating vascular development by short-range intercellular communication (Fukuda et al., 2007). Peptides are often referred to as proteins smaller than 100 amino acids and derived from precursor proteins or directly translated from short open reading frames embedded in longer transcripts (Tavormina et al., 2015). The CLAVATA3/EMBRYO SURROUNDING REGION-RELATED (CLE) peptides are essential for plant development and stress responses (Wang and Fiers, 2010; Tavormina et al., 2015). The Arabidopsis genome contains 32 *CLE* genes encoding 27 unique peptides (Yamaguchi et al., 2016; Fukuda and Hardtke, 2020). These CLE peptides are recognized at the cell surface by transmembrane receptors and activate intracellular signal transduction, therefore regulating plant growth and development (Fletcher, 2020). In this mini-review, we summarize the function and interaction of peptide signaling pathways in vascular development (Table 1) and discuss the gaps in current knowledge and provide perspective for future research.

**Table 1:** CLAVATA3/EMBRYO SURROUNDING REGION-RELATED (CLE) peptides and their receptors in regulating vascular development.

Peptide	Receptors	Function
CLE41/44	PXY/TDR and SERKs	Cambial cell division, xylem differentiation, and vascular patterning
EPFL4/6	ER and ERL1	Vascular cambial cell division
CLE9/10	BAMs	Xylem differentiation
CLE25	CLV2-CLERK	Phloem initiation
CLE45	BAM3, CLV2-CRN	Phloem cell differentiation

### Vascular Development

**Figure 1:**
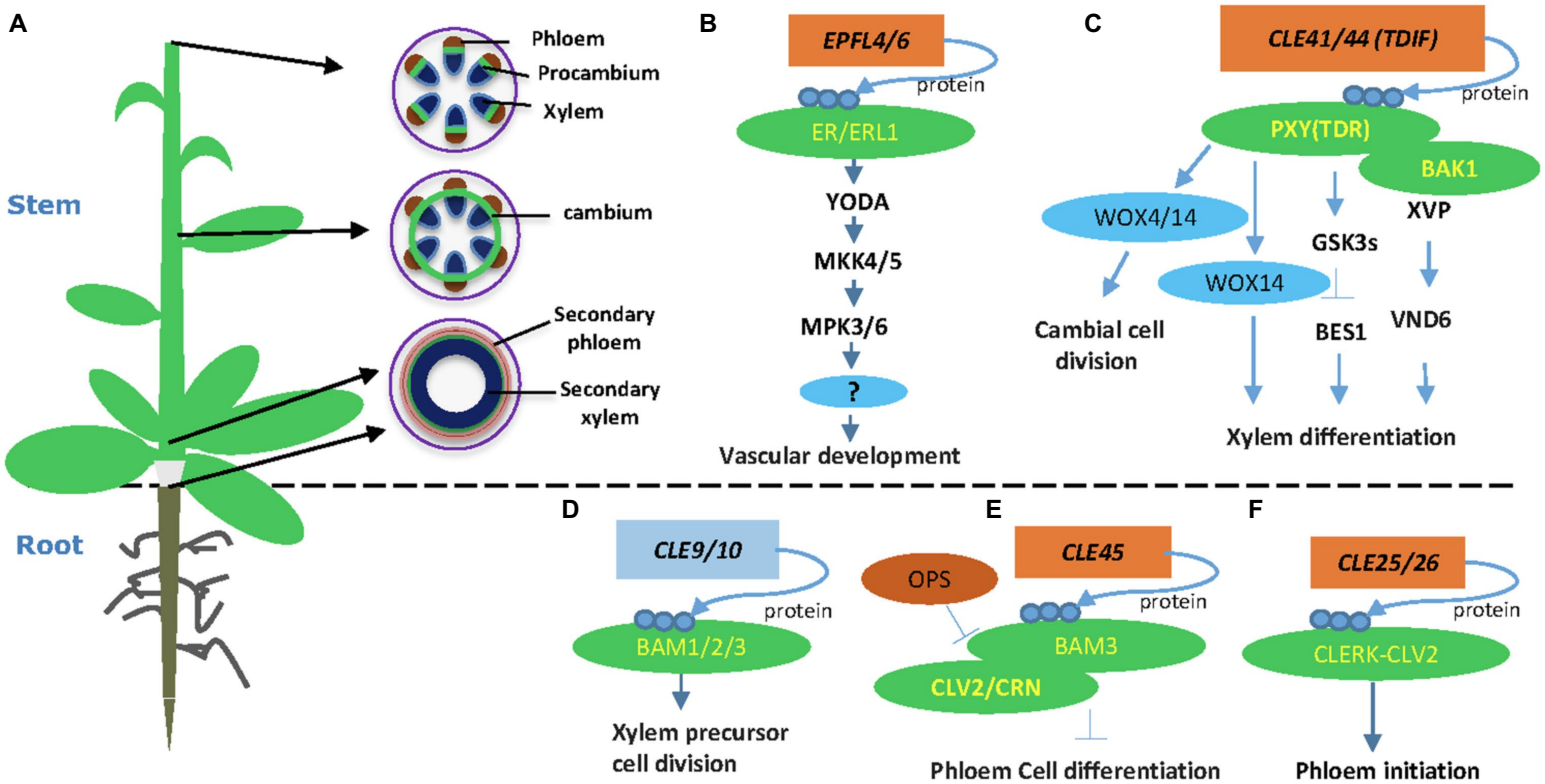
Plant-specific CLE and Epidermal Patterning Factor-Like (EPFL) peptide signaling pathways in vascular development. **(A)** An Arabidopsis plant and different stages of vascular developmental in the stem. A young stem develops discrete vascular bundles that are comprised of phloem, xylem, and intervening procambium. In developing stems, cambial cells from vascular and interfascicular regions form a closed ring structure. Secondary growth produces secondary phloem and secondary xylem in both mature stem and hypocotyls. **(B)** The EPFL4 and EPFL6 peptides regulate stem vascular development in Arabidopsis. **(C)** CLE41/44 regulates vascular cambial cell division and xylem differentiation in the stem. **(D)** The CLE9/10 peptide regulates xylem formation. **(E)** The CLE45-BAM3 peptide inhibits root protophloem cell differentiation. **(F)** The CLE25 peptide promotes root protophloem cell differentiation. Arrows depict positive regulatory relationships and bars depict negative regulatory relationships.

Plant vasculatures transport water and nutrients to all developing organs. In the Arabidopsis stem, the vascular tissues are organized as bundles, in which the procambium or cambium resides in the middle, xylem inside, and phloem outside of the bundle (Figure 1A). At the shoot tip, vascular bundles are well separated along the stem periphery (Figure 1A). In the more mature stem, cambium starts developing in positions between bundles, connects vascular cambium, and forms a ring of cambial cells (Figure 1A). At the bottom of the stem, cambium cells divide and form xylem precursor cells inward and phloem precursor cells outward of the stem. Eventually, these precursors differentiate into secondary xylem and secondary phloem, respectively (Figure 1A). Other plant organs, such as the root and hypocotyls, also go through secondary growth depending on the activity of cambium cells (Wang, 2020). Regulation of vascular development is vital for plant secondary growth and crop production and therefore remains a critical research area in plant biology.

### TDIF-PXY Signaling Regulates Vascular Development

The proliferation of cambial stem cells is essential for vascular development and the mechanical strength of the plant stem. Peptide signals play crucial roles in regulating vascular development through cell-to-cell communication (Fukuda and Hardtke, 2020). The tracheary element differentiation inhibitory factor (TDIF) peptide is so far the best-understood peptide in this process (Fletcher, 2020; Wang, 2020). Tracheary element differentiation inhibitory factor is synthesized from the phloem and travels to the cambium, where TDIF binds to its receptor PHLOEM INTERCALATED WITH XYLEM/TDIF RECEPTOR (PXY/TDR) on the plasma membrane (Hirakawa et al., 2008; Etchells et al., 2016). Overexpression of TDIF coding genes *CLE41* and *CLE44* or exogenous application of synthetic TDIF peptide promote procambial cells proliferation and inhibit xylem cell differentiation (Hirakawa et al., 2008; Whitford et al., 2008; Etchells and Turner, 2010). These experiments established the function of the TDIF-PXY module in cambial cell proliferation, xylem differentiation, and vascular patterning (Hirakawa et al., 2008; Etchells and Turner, 2010). Downstream of the TDIF-PXY module is two WUSCHEL-related HOMEOBOX (WOX) transcription factors, WOX4 and WOX14 (Hirakawa et al., 2010; Etchells et al., 2013; Wang et al., 2013). WOX4 is expressed mainly in the procambium and cambium, and its expression level is upregulated by applying CLE ligand (Hirakawa et al., 2010). WOX14 acts redundantly with WOX4 in regulating vascular cell division (Etchells et al., 2013). The TDIF-PXY-WOX signaling plays a crucial role in the maintenance of the vascular meristem during secondary growth (Hirakawa et al., 2010; Yamaguchi et al., 2016).

The crystal structure of the extracellular domain of PXY/TDR in complex with TDIF peptide has been determined (Morita et al., 2016; Zhang et al., 2016a). The extracellular domain of PXY/TDR adopts a super-helical structure comprising 22 leucine-rich repeats (LRRs) and specifically recognizes TDIF by its inner concave surface (Morita et al., 2016). The TDIF peptide mainly adopts a “Ω”-like conformation binding the inner surface of the LRRs (Zhang et al., 2016a). The highly conserved amino acids of TDIF, including Hyp4, G6, Hyp7, and P9, are critical for the interaction with PXY/TDR (Zhang et al., 2016a). However, there are some discrepancies regarding which amino acids of PXY/TDR are essential to the interaction with TDIF (Morita et al., 2016; Zhang et al., 2016a). Structure-based sequence alignment showed that the TDIF-interacting motifs are conserved among plant species, suggesting a conserved TDIF-PXY recognition mechanism (Morita et al., 2016).

The TDIF-PXY-WOX4 signaling is conserved in plants. The TDIF peptide was first discovered from an analysis in zinnia (Ito et al., 2006). Later, TDIF peptide was found in Arabidopsis (Hirakawa et al., 2008) and the genomes of Euphyllophytes (Hirakawa and Bowman, 2015). The function of TDIF-PXY-WOX4 signaling has been investigated in poplar. Transgenic studies showed that the tissue-specific expression of *PttPXY* and *PttCLE41* genes resulted in enhanced secondary growth in hybrid aspen, indicating that TDIF-PXY signaling is conserved in poplar (Etchells et al., 2015). Consistent with this study, *PttWOX4* gene is specifically expressed in the cambial region and controls cell division activity in the vascular cambium (Kucukoglu et al., 2017).

In a recent study, a regulatory network downstream of TDIF-PXY was identified using an enhanced yeast one-hybrid system (Smit et al., 2020). Among the transcription factor-promoter interactions, WOX14 positively regulated the expression of *TARGET OF MONOPTEROS 6* (*TMO6*), which encodes a Dof-type transcription factor (Schlereth et al., 2010). In addition, WOX4 and TMO6 both positively regulate the expression of a third transcription factor gene, *LATERAL ORGAN BOUNDARIES DOMAIN4* (Smit et al., 2020). These data support a mechanism that this feed-forward loop controls vascular stem cell proliferation and helps to define the function of PXY signaling in determining the shape of the vascular bundle (Fletcher, 2020; Smit et al., 2020).

The TDIF-PXY signaling module regulates cell division in the (pro-)cambium and controls vascular organization *via* interacting with other factors (Etchells et al., 2016). The SOMATIC EMBRYOGENESIS RECEPTOR KINASE (SERK) family regulates plant growth and development, as well as many other biological processes (Albrecht et al., 2008). The SERK genes are expressed in procambial cells, suggesting that the SERK family may have a function in regulating vascular development (Kwaaitaal and De Vries, 2007). Indeed, the TDIF receptor PXY forms heterodimers with SERKs, including SERK1, SERK2, and SERK3 (BAK1), in regulating procambial cell proliferation (Zhang et al., 2016b). A NAC domain transcription factor XVP is localized on the plasma membrane and forms a complex with TDIF co-receptor PXY-BAK1 (Yang et al., 2020a). The XVP is expressed in the cambium and regulates xylem differentiation and vascular patterning (Yang et al., 2020a). Further study showed that XVP regulates xylem differentiation through a crucial factor VASCULAR-RELATED NAC-DOMAIN6. The XVP promotes the expression of TDIF-encoding gene *CLE44*, whereas TDIF negatively regulates the expression of *XVP*, suggesting a feedback regulating loop (Figure 1). However, it is still not clear whether and how XVP proteins are translocated to the nucleus.

### EPFL4/6-ER Signaling in the Regulation of Vascular Cambium

The EPIDERMAL PATTERNING FACTOR-LIKE 4 (EPFL4) and EPFL6/CHALLAH (CHAL) secretory peptides were first identified as regulators of inflorescence development through activating the ER signaling (Uchida et al., 2012). Two additional ER paralogs, ER-LIKE 1 (ERL1) and ERL2, together with ER, play crucial roles in regulating inflorescence architecture, organ shape, and epidermal stomatal patterning in *Arabidopsis* (Shpak et al., 2004; Uchida et al., 2012). In vascular development, the endodermis-produced EPFL4 and EPFL6 peptides function as ligands for phloem-located ER in regulating procambial cell division and stem elongation (Fischer and Teichmann, 2017; Ikematsu et al., 2017). Therefore, the cell-to-cell communication between the endodermis and the phloem plays a crucial role in procambial development (Ikematsu et al., 2017). Both *ER* and *ERL1* are expressed in inflorescence stem and function redundantly in regulating procambial development and xylem differentiation (Ikematsu et al., 2017; Tameshige et al., 2017). The *mpk3/mpk6* and mkk*4/mkk5* mutants showed short pedicels and a corymb-like inflorescence similar to the phenotypes of the *er* mutant (Meng et al., 2013). Further evidence showed that the MAPK cascade functions downstream of the ER receptor in regulating pedicel length and inflorescence architecture (Meng et al., 2013). Therefore, the MAPK cascade, involving YDA-MKK4/MKK5-MPK3/MPK6, functions downstream of ER in regulating cell proliferation and plant organ development (Figure 1). It would be interesting to investigate if this MAPK pathway also regulates procambium development in the stem and hypocotyl.

### Other CLE Peptide Signaling Pathways in Vascular Development

Besides CLE41/CLE44, EPFL4, and EPFL6, other peptides, including CLE9/10, CLE25, and CLE45, also play critical roles in vascular development (Fukuda and Hardtke, 2020). The *CLE9* and *CLE10* genes are preferentially expressed in xylem precursor cells of the root vasculature (Kondo et al., 2011). Interestingly, the peptide CLE9/10 forms a complex with the receptor kinase HAESA-LIKE 1 (HSL1) to regulate stomatal lineage cell division. In the root xylem development, CLE9/10 is perceived by BARELY ANY MERISTEM (BAM) class receptor kinases in regulating periclinal cell division (Figure 1; Qian et al., 2018). BARELY ANY MERISTEM 1 and its homologs, BAM2 and BAM3, are class XI LRR receptor-like kinases similar to CLV1 and PXY (DeYoung et al., 2006). The *bam1bam2* and *bam1bam3* mutants exhibited increased xylem files similarly to the *cle9* mutant (Qian et al., 2018). Indeed, physical interaction between CLE9/10 and BAM1 was demonstrated by three independent approaches (Shinohara et al., 2012; Anne et al., 2018; Qian et al., 2018), confirming the BAMs as CLE9 receptors in root xylem development.

The CLE45 peptide forms a complex with BARELY ANY MERISTEM 3 (BAM3) to regulate Arabidopsis protophloem differentiation (Depuydt et al., 2013). Protophloem consists of narrow thin-walled cells and is developed from procambium. Studies revealed that CLE45 is expressed from protophloem sieve elements in root meristems and perceived fully by CLAVATA 2 (CLV2) and the CORYNE (CRN) receptor proteins (Hazak et al., 2017). CRN is required for BAM3-mediated CLE45 signaling *via* stabilizing BAM3 expression. In addition, a protophloem-specific CRN expression rescues the resistance of the *crn* mutant to root-active CLE peptides (Hazak et al., 2017). Collectively, these data support a vital function of the CLE45 and CLV/BAM receptors in vascular development (Hazak et al., 2017). Downstream of BAM3, a MEMBRANE-ASSOCIATED KINASE REGULATOR protein, positively regulates CLE45 signaling (Kang and Hardtke, 2016). Further, OCTOPUS (OPS), a polarly localized membrane-associated protein, promotes root protophloem differentiation by antagonizing CLE45 signaling through direct interference with BAM3 and CLV2-CRN (Figure 1; Truernit et al., 2012; Rodriguez-Villalon et al., 2014; Breda et al., 2019).

The CLE25 and CLE26 play crucial roles in regulating root protophloem development (Czyzewicz et al., 2015; Anne et al., 2018; Ren et al., 2019). CLE25 expression is tightly associated with phloem initiation, and the loss-of-function mutant *cle25* shows a delayed protophloem differentiation phenotype (Ren et al., 2019). Genetic screening for suppressors identified CLV2 as a potential receptor for CLE25 (Ren et al., 2019). Further peptide assay and protein interaction experiment found that CLE-RESISTANT RECEPTOR KINASE (CLERK) interacts with CLV2 and possibly forms a receptor complex for CLE25 (Ren et al., 2019). However, direct binding between CLE25 and the CLERK-CLV2 receptor complex remains to be established experimentally.

The CLE45 and CLE25/26 peptides are all expressed in root and repress phloem development as shown by expression analyses and synthetic peptides treatment experiments (Kinoshita et al., 2007; Jun et al., 2010). CLE25 may be involved in the formative cell division during protophloem differentiation. It is still unclear which division step is regulated by CLE25 in the two successive formative cell division steps during protophloem development (Rodriguez-Villalon et al., 2014; Ren et al., 2019). In addition, there are some differences in terms of perception of these peptides. The CLE25 and CLE26 peptides could be perceived by CLERK (Anne et al., 2018; Ren et al., 2019), while CLE45 is perceived by BAM3 (Kang and Hardtke, 2016; Hazak et al., 2017). Further, CLE25 may be involved in response to detrimental environmental signals (Takahashi et al., 2018). Further investigation is needed to clarify the functional difference of these peptides in protophloem development.

### The Crosstalk Between TDIF-TDR Signaling and Other Signaling Pathways

The development of vascular tissue involves interactions between TDIF-PXY and other peptide signaling pathways. The receptor kinases PXY and ER genetically interact to coordinate cell division and organization (Uchida and Tasaka, 2013; Etchells et al., 2016; Wang et al., 2019). Gene expression analysis suggested that *PXL1* and *PXL2* expression is upregulated in *er* mutant stems (Wang et al., 2019). The expression of *EPFL4* and *EPFL6* is altered in *pxf* (mutation of the PXY family, including *pxy*, *pxl1*, and *pxl2*) and *er pxy* mutants (Wang et al., 2019). Further study showed that the mutant of *er pxf* had considerably fewer cells per vascular bundle than either *pxf*, *er*, or *pxy* er, suggesting that *PXL1* and *PXL2* are functionally redundant with *ER* to regulate vascular proliferation in the stem (Wang et al., 2019). These results demonstrate that PXY and ER coordinately regulate vascular proliferation and organization *via* inter-tissue signaling (Wang et al., 2019).

TDFI-PXY signaling also interacts with plant hormonal signals, such as brassinosteroids (BR), auxin, and ethylene signaling. The GLYCOGEN SYNTHASE KINASE 3 (GSK3) family members, including BRASSINOSTEROID-INSENSITIVE 2 (BIN2), are crucial downstream components of the TDIF signaling pathway in suppressing xylem differentiation (Kondo et al., 2014). The PXY/TDR receptor interacts with GSK3s on the plasma membrane and activates GSK3s in a TDIF-dependent fashion (Kondo et al., 2014). The interaction between PXY/TDR and BIN2 inhibits the transcription factor BES1 to prevent procambial cell differentiation into the xylem (Kondo et al., 2014). Recently, an additional study showed that the GSK3 protein BIN2-LIKE 1 (BIL1) integrates the PXY/TDR signaling through phosphorylating the auxin response factor MP/ARF5, which limits the effect of MP/ARF5 on ARR7 and ARR15 expression, thus enhancing vascular cambial activity (Han et al., 2018). Together, these results suggest that BIL1 mediated the crosstalk between peptide signaling and hormone signaling to maintain the cambial activity (Figure 1; Han et al., 2018). Ethylene promotes cambium activity (Love et al., 2009; Yang et al., 2020b) and interacts with TDIF-PXY signaling (Etchells et al., 2012). The interaction between TDIF-PXY and plant hormone may help plants better perceive and respond to long-distance developmental and environmental signals.

### The Crosstalk Between EPFL4/6-ER Signaling and Other Signaling Pathways

The LRR receptor-like kinase ER is a stem growth regulator and involves in diverse developmental processes, including epidermal and mesophyll development, stomatal density, and vascular development (Tameshige et al., 2017). For example, hydrogen peroxide induces cortex proliferation, while SPINDLY, an O-linked glucosamine acetyltransferase, functions downstream of ER to fine-tune cortex proliferation, indicating a connection between ER signaling and cellular redox homeostasis (Cui et al., 2014). Another study showed that an LRR receptor-like kinase SUPPRESSOR OF BIR1-1, previously known to control plant immune responses and abscission, appears downstream of BREVIPEDICELLUS (BP) and ER to regulate the precocious differentiation of xylem fiber (Milhinhos et al., 2019). In addition, ER signaling pathway and the SWR1 chromatin remodeling complex jointly regulate inflorescence architecture by promoting the expression of the PACLOBUTRAZOL RESISTANCE (PRE) gene family (Cai et al., 2017). Further study showed that a growth-related transcription factor HOMOLOG OF BEE2 INTERACTING WITH IBH1 (HBI1) functions downstream of PRE1 to regulate inflorescence architecture by promoting pedicel elongation (Cai et al., 2021). These studies indicate potential crosstalks between ERFL4/6 and other pathways in regulating vascular development.

## Conclusion

Significant progress has been made in our understanding of peptide signaling, including the TDIF-PXY/TDR module and the EPFL4/6–ER/ERL1 module, in regulating vascular development (Fukuda and Hardtke, 2020; Wang, 2020; Wang and Wang, 2020). While the TDIF-PXY signaling in vascular development has been the subject of much research since the discovery of TDIF, many questions remain. For instance, compared to the wild type, *pxy* mutants showed more fiber cells indicating that PXY has a potential function in regulating xylem development (Fisher and Turner, 2007). Moreover, environmental factors, including light, carbon dioxide concentration, and drought, all affect the expression pattern of several EPFL family genes, indicating that ER signaling could respond to external environmental stimuli (Richardson and Torii, 2013). Therefore, studying the crosstalk between environmental factors and peptide signaling in vascular development is an exciting field in the future.

Further, the EPFL4/6–ER/ERL1 module regulates procambial maintenance in inflorescence stems (Uchida et al., 2012), and a MAPK signaling module YDA-MKK4/5-MPK3/6 functions downstream of ER (Meng et al., 2013). Identification of the substrates of MPK3/6 would be crucial to understand the molecular mechanism of the EPFL-ER signaling. The crosstalk between TDIF-PXY and EPFL-ER signaling has been proposed, but the mechanistic interaction has not been established. It is possible that a common downstream signaling hub may connect both pathways in regulating cambial activity.

In addition, the BAM1 receptor can potentially bind both CLV3 and CLE25 peptides, indicating that the same receptors can perceive different CLE peptides in various biological contexts (Takahashi et al., 2018; Fletcher, 2020). In contrast, the CLE9/10 peptide ligand is recognized by two different receptor kinases HSL1 and BAM3 in regulating stomatal lineage cell division and periclinal cell division of xylem precursor cells, respectively (Qian et al., 2018). Therefore, the same receptor can perceive different CLE peptides, while the same peptide can bind to different receptors, indicating extremely complex peptide signal transduction networks in plants. The complexity may be necessary for peptide signaling to have developmental and organ-specific functions. Further dissection of the peptide signaling pathways will provide new insight into our understanding of vascular development.

## Author Contributions

BY and HW wrote the review. All authors contributed to the article and approved the submitted version.

## Conflict of Interest

The authors declare that the research was conducted in the absence of any commercial or financial relationships that could be construed as a potential conflict of interest.

## Publisher’s Note

All claims expressed in this article are solely those of the authors and do not necessarily represent those of their affiliated organizations, or those of the publisher, the editors and the reviewers. Any product that may be evaluated in this article, or claim that may be made by its manufacturer, is not guaranteed or endorsed by the publisher.
